# Severe Proximal Celiac Trunk Stenosis Without Significant Aortic Atherosclerosis: A Case Report

**DOI:** 10.7759/cureus.96212

**Published:** 2025-11-06

**Authors:** Mahfuza A Khan, Mohammed S Alam, Roxana Lazarescu, Argavan Ansari, Srikar Darsi, Shriya Patel

**Affiliations:** 1 Internal Medicine, Sylhet MAG Osmani Medical College, Sylhet, BGD; 2 Medicine and Surgery, Wyckoff Heights Medical Center, New York, USA; 3 Internal Medicine, Wyckoff Heights Medical Center, New York, USA; 4 Internal Medicine, Touro College of Osteopathic Medicine, New York, USA; 5 Internal Medicine, Xavier University School of Medicine, Oranjestad, ABW

**Keywords:** celiac trunk stenosis, ct angiography, median arcuate ligament syndrome, nonspecific abdominal pain, weight loss

## Abstract

A 76-year-old male with a history of gastritis, gastric ulcer perforation, hypertension, and active smoking presented with recurrent chest pain, abdominal pain, and back pain. Workup excluded acute coronary syndrome, pulmonary embolism, aortic dissection, and mesenteric ischemia. CT angiography revealed severe proximal celiac trunk stenosis, an ectatic descending thoracic aorta, bilateral renal cysts, emphysema, and a hepatic hypodensity. Further evaluation demonstrated non-obstructive coronary disease, possibly related to endothelial dysfunction. This case highlights the diagnostic complexity of back pain and chest pain in elderly patients with other comorbidities, emphasizing the importance of distinguishing incidental vascular findings from clinically significant pathology. A multidisciplinary approach involving cardiology, vascular surgery, gastroenterology, pulmonology, and nutrition services was required to optimize care.

## Introduction

The celiac trunk supplies blood to the stomach, liver, spleen, and pancreas. Proximal narrowing most commonly results from atherosclerotic disease or compression by the median arcuate ligament; less frequent causes include congenital or inflammatory conditions [1,2]. Many individuals with radiographic stenosis remain asymptomatic, complicating clinical decision-making. This case is novel because it demonstrates a clear correlation between imaging metrics and the underlying pathophysiology of dynamic celiac trunk compression.

When clinically significant, stenosis may contribute to mesenteric ischemia, alter outcomes after abdominal surgery, or rarely regress spontaneously [3,4]. Modern imaging, particularly CT angiography and duplex ultrasound, remains central for diagnosis and for assessing collateral circulation [5].

We report a case of severe proximal celiac trunk stenosis in an elderly patient with extensive comorbidities but without significant aortic atherosclerosis, outlining diagnostic challenges and management considerations.

## Case presentation

A 76-year-old male with a history of gastritis, gastric ulcer perforation surgically repaired in the 1980s, and hypertension presented with five hours of diffuse back pain radiating to the chest and epigastric region. The pain was squeezing in nature, rated 4/10, and not associated with nausea, vomiting, diaphoresis, syncope, or palpitations. He denied gastrointestinal bleeding, respiratory symptoms, fever, or neurologic complaints.

He was an active smoker (½ pack per day), consumed alcohol socially, and denied illicit drug use. On presentation, he was hemodynamically stable and in no acute distress. The abdominal exam was benign. Physical exam was only notable for cachexia (BMI 16.2 kg/m^2^). EKG showed sinus rhythm with nonspecific lateral T wave changes, and serial troponins were negative.

**Figure 1:**
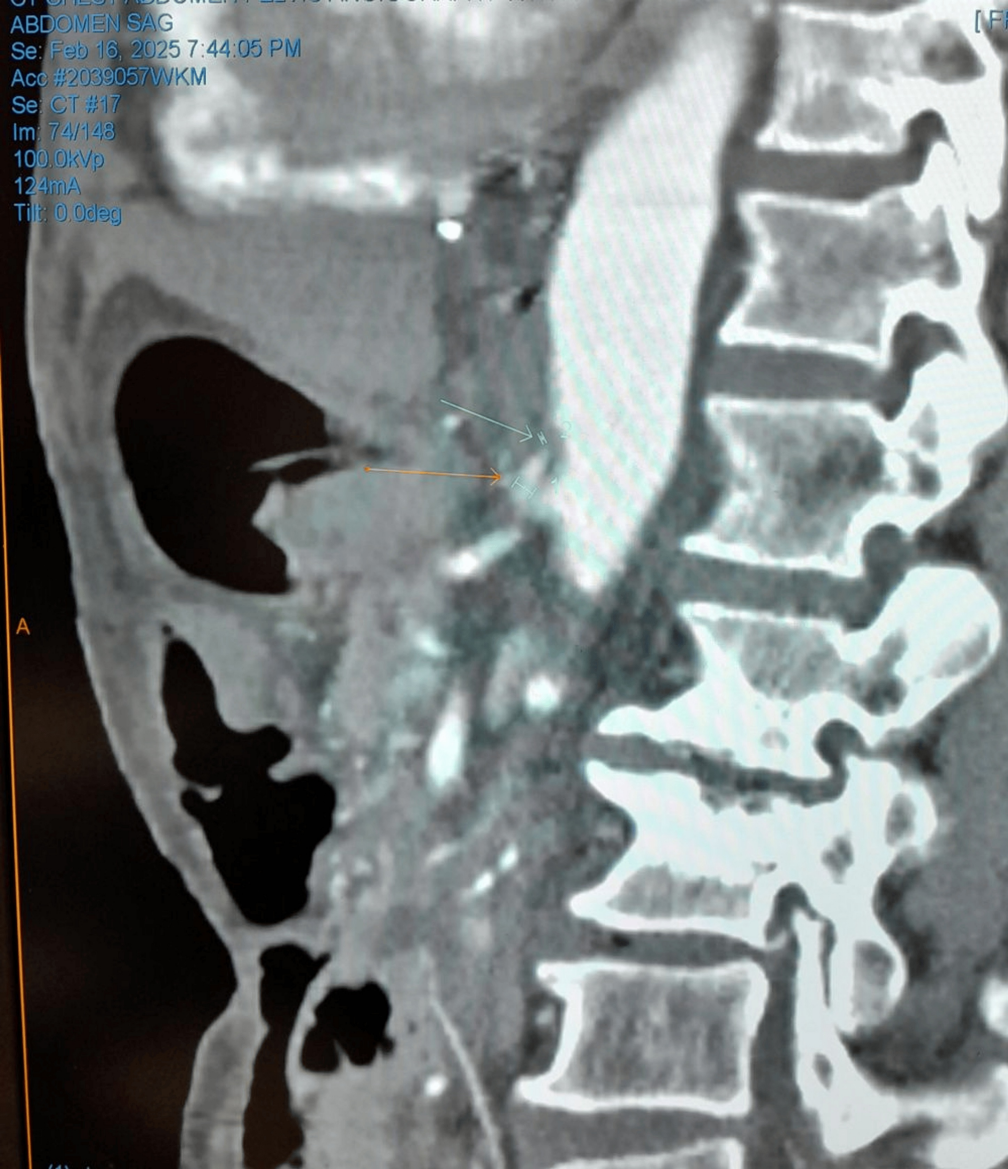
CT angiography of the chest, abdomen, and pelvis, with the green arrow showing severe proximal celiac trunk stenosis and the yellow arrow showing post stenosis segment of the celiac trunk.

**Figure 2:**
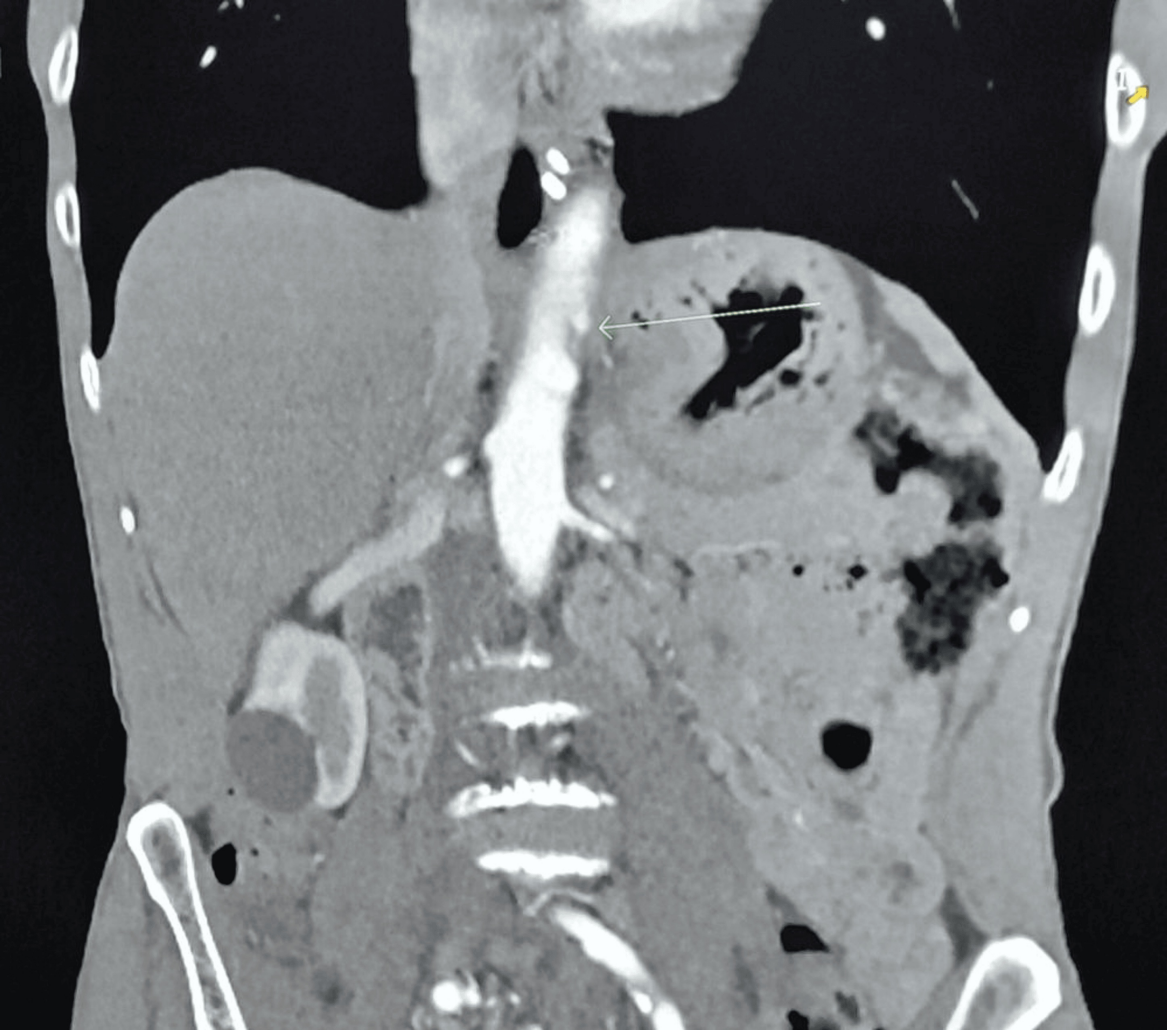
CT angiography of the chest, abdomen, and pelvis, with the green arrow showing severe proximal celiac trunk stenosis.

CT angiography of the chest, abdomen, and pelvis revealed no pulmonary embolism or aortic dissection. Findings included moderate to severe emphysema, an ectatic descending thoracic aorta measuring 3.1 cm, severe proximal celiac trunk stenosis with the diameter of 0.06 cm and post stenosis segment with the diameter of 0.4 cm, bilateral renal cysts up to 5 cm, fatty infiltration of the liver, and a 0.8 cm hypodense right hepatic lobe lesion, favored to be a cyst (Figures 1, 2). Laboratory studies, including a complete blood count, metabolic panel, and thyroid-stimulating hormone (1.844 mIU/L), were unremarkable (Table 1). 

**Table 1:** Lab values TSH: thyroid-stimulating hormone, LDL: low-density lipoprotein

Parameters	Patient values	Reference range
Troponin, set -1	6.7 ng/L	3.0–58.9 ng/L
Troponin, set - 2	6.9 ng/L	3.0–58.9 ng/L
TSH	1.844 MIU/L	.358–3.74 MIU/L
Lipase	24 U/L	13–75 U/L
LDL cholesterol	97 MG/DL	1–100 MG/DL

He was admitted for telemetry monitoring to rule out acute coronary syndrome and was started on aspirin and atorvastatin as per cardiology recommendation. Transthoracic echocardiography demonstrated a left ventricular ejection fraction of 55-60% without wall motion abnormalities or diastolic dysfunction.

Vascular surgery evaluated the patient for severe celiac stenosis. Given the absence of postprandial abdominal pain, food aversion, and a benign abdominal examination, the stenosis was considered incidental. No acute intervention was required, and outpatient follow-up was advised.

Consultations were arranged with gastroenterology for the hepatic lesion, urology for renal cysts, and pulmonology for emphysema. The patient was counseled regarding smoking cessation. He remained clinically stable during hospitalization and was discharged home with instructions for close outpatient follow-up.

## Discussion

Celiac artery stenosis is an uncommon vascular finding that may be asymptomatic or present with mesenteric ischemia, depending on the degree of obstruction and collateral blood flow. The celiac artery’s role in supplying the upper abdominal viscera makes stenosis clinically relevant, even when detected incidentally [1].

Collateral circulation via the superior mesenteric artery and pancreaticoduodenal arcades often maintains perfusion, explaining why many patients remain symptom-free [2,3]. However, individuals with advanced vascular disease or extrinsic compression (e.g., median arcuate ligament) may develop ischemic symptoms such as postprandial pain, nausea, or weight loss [4-6].

Cross-sectional imaging is indispensable for diagnosis. CTA and Doppler ultrasound confirm stenosis and assess collateral development, while often revealing incidental findings [2]. Recognition is crucial, as progressive stenosis may predispose to mesenteric ischemia, aneurysms of collateral vessels, or complications during hepatobiliary and pancreatic surgery [4].

Management depends on the clinical context. Asymptomatic patients are often monitored conservatively [5], while symptomatic or progressive cases may require revascularization or decompression via surgical or endovascular approaches [6,7].

In our patient, non-interventional management was chosen due to severe malnutrition, advanced COPD, and frailty. The coexisting non-obstructive coronary artery disease complicated the evaluation of chest pain episodes, while persistent hypercalcemia and nutritional deficits emphasized the importance of a multidisciplinary approach.

This case is notable because the stenosis occurred without diffuse aortic atherosclerosis, a rare finding. The decision for conservative management highlights how frailty, comorbidities, and overall risk often outweigh anatomical findings in clinical decision-making.

## Conclusions

Severe celiac trunk stenosis can remain clinically silent due to collateral circulation. However, it has important implications in frail patients with multiple comorbidities. In this patient, conservative management was appropriate given his poor nutritional state and pulmonary disease. This case highlights the rarity of severe stenosis without aortic atherosclerosis and underscores the importance of individualized, multidisciplinary care.
